# Active contact tracing beyond the household in multidrug resistant tuberculosis in Vietnam: a cohort study

**DOI:** 10.1186/s12889-019-6573-z

**Published:** 2019-02-28

**Authors:** Thi Thanh Thuy Hoang, Viet Nhung Nguyen, Ngoc Sy Dinh, Guy Thwaites, Thu Anh Nguyen, H. Rogier van Doorn, Frank Cobelens, Heiman F. L. Wertheim

**Affiliations:** 1 National Tuberculosis Control Programme of Vietnam- National Lung Hospital (VNTP-NLH), Hanoi, Vietnam; 2 Vietnam Association for Tuberculosis and Lung Disease, Hanoi, Vietnam; 3 Department of Global Health and Amsterdam Institute for Global Health and Development, Academic Medical Center, Amsterdam, Netherlands; 4 Oxford University Clinical Research Unit, Ho Chi Minh City, Vietnam; 5 Woolcock Institute Of Medical Research, Hanoi, Vietnam; 6 Oxford University Clinical Research Unit, Hanoi, Vietnam; 7 Nuffield Department of Clinical Medicine, Centre for Tropical Medicine, University of Oxford, Oxford, UK; 8 Department of Medical Microbiology, Radboudumc, Nijmegen, Netherlands

**Keywords:** Multi drug resistance tuberculosis, Contact tracing, Vietnam

## Abstract

**Background:**

Currently in Vietnam contact tracing for multidrug-resistant tuberculosis (MDR-TB) entails passive case finding among symptomatic household contacts who present themselves for diagnosis. Close contacts of MDR-TB cases are therefore not identified adequately. We assessed the added value of active contact tracing within and beyond households using social network questionnaires to identify close contacts of MDR-TB patients in Vietnam.

**Methods:**

We conducted a cohort study using social network questionnaires in which contacts were identified by MDR-TB patients, including contacts from ‘high risk’ places like work. Contacts of MDR-TB patients were followed up and screened over a period of at least 6 months. This included two active screenings and any unscheduled passive screening of self-referred contacts during the study period.

**Results:**

Four hundred seventeen contacts of 99 index cases were recruited, 325 (77.9%) and 160/417 (38.4%) contacts participated in the first and second screenings, respectively. The first screening detected one TB case but the bacteria were not MDR. From passive screening, a household contact was diagnosed with TB meningitis but not through our active approach.

Social network analysis showed that only 1/17 (5.9%) high-risk places agreed to cooperate and were included in the screening, and no MDR-TB cases were detected. There were two pairs of index cases (identified separately) who were found to be contacts of each other and who had been diagnosed before the study started.

**Conclusions:**

No new MDR-TB cases were detected using social network analysis of nearly 100 MDR-TB index cases, likely due to a relatively short follow up time, and loss to follow up (lack of cooperation from contacts or high risk places and lack of available resources in the National Tuberculosis Control Programme).

**Electronic supplementary material:**

The online version of this article (10.1186/s12889-019-6573-z) contains supplementary material, which is available to authorized users.

## Background

The emergence of resistance to anti-tuberculosis drugs, and particularly of multidrug-resistant tuberculosis (MDR-TB) is a serious public health threat and an obstacle to effective global TB control [1]. It is crucial to identify more MDR-TB cases at an earlier stage and provide optimal treatment. Vietnam is ranked 13th among 30 high burden MDR-TB countries (based on estimated incidence by absolute number) with an estimated 5500 MDR-TB among a total of 100.000 notified TB cases per year [2]. Despite the efforts to utilize rapid test to intensify case finding of MDR-TB; in Vietnam, the proportion of MDR-TB cases detected and treated annually is low compared with the estimated number of incident MDR-TB cases (less than 50%, see Additional file 1 for notification and enrollment of MDR-TB cases) [3].

Contact screening of MDR-TB patients is highly recommended by the World Health Organization (WHO) [4]. However, contact investigation of household members only is not sufficient to identify all MDR-TB cases due to transmission outside the household. In rural Vietnam only 1% of index TB patients had a positive household member and 83% of these household TB cases were infected with an isolate that differed from that of their household members [5]. These results are similar to those in higher incidence settings in South Africa, and Malawi [6, 7]. The WHO also recommends to conduct contact investigation beyond the household for patients with MDR-TB and extensively drug-resistant TB (XDR-TB), and to collect additional information regarding their residence and other social settings where transmission may have occurred such as hotels, shelters and bars [4]. Contact tracing using social network questionnaires is a more comprehensive approach than household contact tracing, which includes the linking person to person or person to place for contact investigation [8, 9].

Although screening of close contacts of MDR-TB patients is recommended by the National Tuberculosis Control Programme (NTP) of Vietnam (see Additional file 1 for policy recommended by the NTP Guidelines) [10], there is no system in place to support this. Currently a passive case finding approach is used, where household contacts are advised to seek TB diagnosis when symptomatic.

We assessed the added value of active contact tracing within and beyond the household using social network questionnaires (SNQ) among contacts of MDR-TB patients in Vietnam.

## Methods

### Study design and setting

A cohort study was conducted to analyze the added value of an active screening using SNQ, the questionnaire revealed the patient’s contacts through their social network including the frequently met people and visited places. Contacts were either named by patients or identified from eligible places. Close contacts of MDR-TB patients were enrolled and followed up over a period of at least 6 months and screened for TB and MDR-TB. Contacts were screened at enrolment, followed by an appointment on completion of the first screening and a reminder at 6 months by telephone for the second screening. During the study period, study participants were asked to make an unscheduled visit to district TB units and contact with the district health coordinators if they had any symptom suggestive of TB. The screening consisted of (i) standardized clinical assessment, (ii) chest X-ray among those who were not presumed to have TB by clinical assessment, and (iii) microbiological testing by Gene Xpert MTB/RIF (Xpert, Cepheid, the United States) for patients with a history or chest X-ray suggestive of TB.

### Study population and definitions

The study involved patients with rifampicin resistant TB and their eligible contacts (all ages), who were named by index patients or identified from eligible places as defined below. The minimum sample size was estimated at 100 patients (see Additional file 2 for sample size calculation).

#### Inclusion criteria

Eligible for enrolment were all patients diagnosed with rifampicin-resistant TB or MDR-TB diagnosed by Xpert or by Genotype MTBDR *Plus* Line Probe Assay (Hain Lifescience Nehren, Germany) who were living in Hanoi and started MDR-TB treatment between October 2013 and April 2015. Their defined contacts (household contacts or contacts outside the household, either named by patients or from eligible places) during the 3 months preceding MDR-TB diagnosis were eligible for enrolment as contacts. Eligible high-risk places were physically enclosed spaces where the MDR-TB index case spent an average of at least 4 h a day for at least 14 days, or a cumulative total average duration of at least 8 h per week for at least 8 weeks in 3 months prior MDR-TB diagnosis. For children who were less than 18 years old, information was obtained from their parents or responsible family members.

### Data collection and analysis

We modified and contextualized a published case report form (CRF) [11], which was then validated by fine tuning the language to make sure the respondents can understand and answer our questions adequately, and used to interview consenting patients (see Additional file 2 for description of modifications to the questionnaire). The following data were collected: demographics, medical history, social network including their contacts and frequently visited places, as well as tracing information (name, address, telephone number). As soon as patients were diagnosed and enrolled for treatment during one to two weeks at the provincial hospital, informed consent was obtained, and the interviews with the patients were conducted by trained TB health care workers. Completed patient CRFs were entered in a central database (CliRes) and reviewed by the study coordinator to identify eligible places and contacts (see Additional file 2 for operational definitions) for screening.

Eligible contacts were registered at district TB units to be followed up by the study team. Eligible places were visited by provincial or district coordinators to obtain informed consent from the place’s legal representative, followed by public announcement to call frequent visitors to come for screening to identify additional contacts. Completed contact CRFs were entered in the central database.

Social network analysis was applied to identify links among MDR-TB index cases, contacts, and places. In the network illustrated by the link among patients, contacts and places, contacts or index cases linked to more than one patient were considered as the source of transmission, hence the centers of the network. The centrality degree of the contacts was measured by the number of patients attached with each contact. In order to determine if the contacts or places were mutual (i.e. named by at least 2 confirmed MDR-TB patients), demographics and information such as address and telephone number of the places and contacts were collected and compared. Mutual contacts or places were identified when identical information was obtained between pairs of contacts or pairs of places named by different patients. The Social network analysis also looked at the density illustrated by how closely contacts and patients are connected. The number of contacts per patient was used to rank the closeness level between patient and contacts [12].

### Statistical analysis

Data were entered in MS Access software (Microsoft Inc., USA) and then transferred to SPSS 16.0 for statistical analysis. Descriptive statistics, including frequency, median, interquartile range (IQR), proportion and 95% confidence intervals (95% CIs), were performed where appropriate. The comparisons were tested statistically using Chi-Square test to compare proportions. *P*-values (2-sided) below 0.05 were considered significant.

## Results

### Characteristic of MDR-TB patients (index cases)

Of 112 eligible patients, 99 were enrolled into the study as MDR-TB index cases. All patients were adult, 51(51%) were 35–54 years old and 77 (78%) were male. Four patients were HIV positive (Table 1).

**Table 1:** Characteristics of patients at baseline

	*n*	%
Total patients	99	100
Characteristic		
Age group		
0–14 years	0	0
15–24 years	14	14
25–34 years	15	15
35–44 years	28	28
45–54 years	23	23
55–64 years	14	14
65 year and above	5	5
Median age	43	
Mean age (sd)	42.3 (13.9)	
Gender		
Male	77	78
Female	22	22
HIV (+)	4	4
Sputum smear		
Negative	23	23
Positive	76	77
Grade among smear positive^*a*^		
Scanty	20	26
1+	33	43
2+	13	17
3+	10	13
Chest X-ray		
Cavity	32	32
Patient category^*b*^		
New	14	14
Non-converters of first line drug for new cases	11	11
Non-converters of first line drug for retreatment cases	1	1
Previously treated cases	69	70
Others	4	4

^a^Based on the International Union Against Tuberculosis and Lung Disease (IUATLD)-recommended grading of sputum smear microscopy results (*n* = 76)

^b^Refer to Additional file 2 for definition of patient category

Sputum smear and chest X-ray were performed for all patients: 76/99 (77%) had a smear- positive result. Thirty-two patients (32%) had X-ray signs of cavitation, all of whom were smear-positive. Seventy patients (71%) had been previously treated with first-line anti-TB drugs. These included patients detected as MDR-TB when starting retreatment or detected later when found to be smear-positive (non-converters) after two months of retreatment. Fourteen patients (14%) had received no or less than one month of TB treatment previously. The remaining 15 patients (15%) included 11 non-converters during their first treatment course and 4 patients previously treated in the private sector with unknown outcome.

### Characteristics of contacts

We identified 496 contacts and 17 high risk places based on information provided in the SNQ: 481 contacts were named by patients and 17 high risk places were approached by visiting for informed consent. Of which 1 place agreed to cooperate and subsequently an additional 15 contacts were identified.

**Fig. 1:**
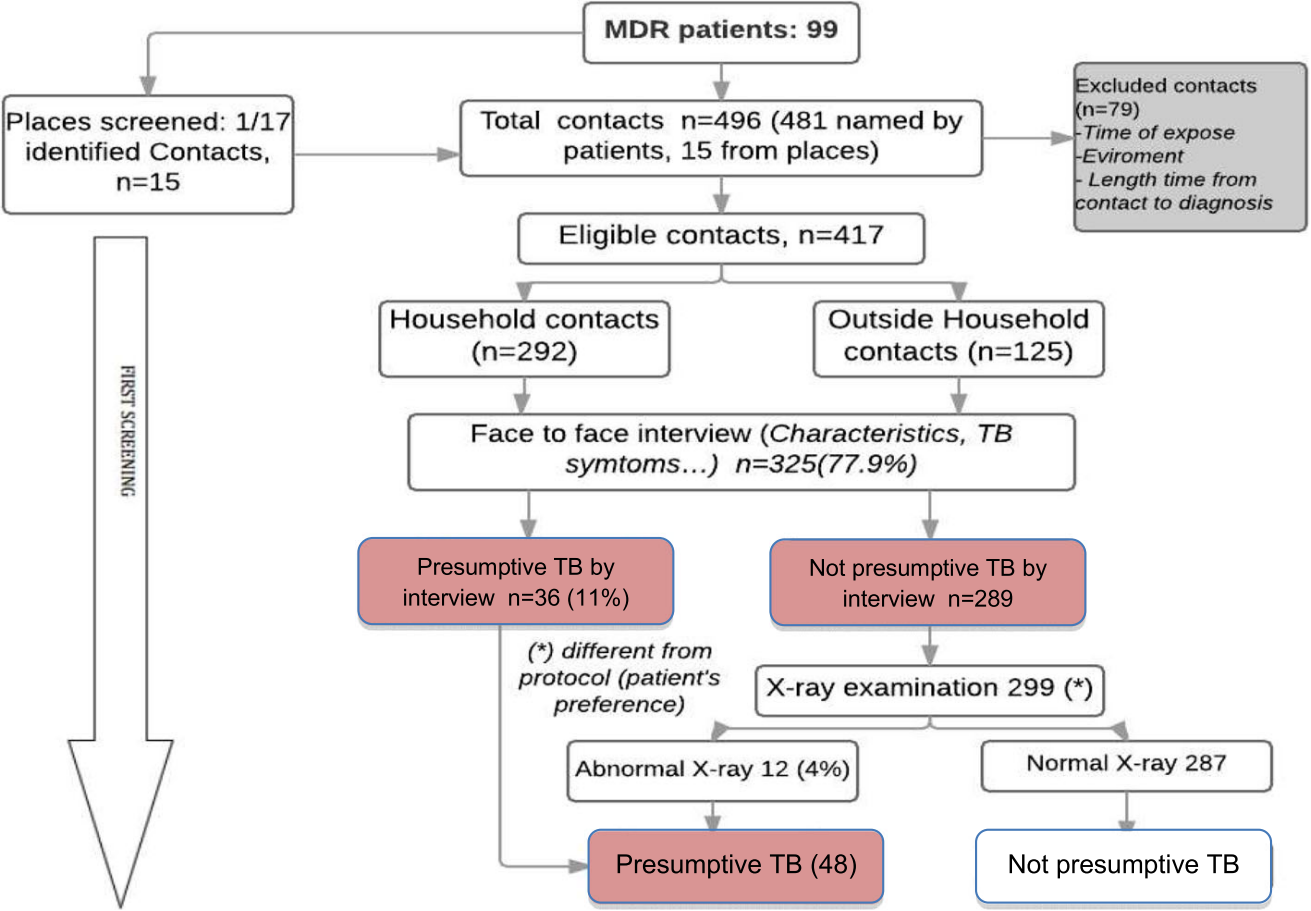
Flow chart of TB screening practice (part A)

Seventy-nine contacts were excluded from the study because they were not living in Hanoi (*n* = 16) and/or had not been in direct contact with the index patient during the 3 months before diagnosis (*n* = 69) (Fig. 1), leaving 417 eligible contacts whose characteristics are described in Table 2. They included 292 (70.0%) household contacts and 125 (30.0%) non-household contacts. Of the 125 non-household contacts, one contact came from a high-risk place, and the others had been named by index patients. Of the 417 eligible contacts, 189 (45.3%) were males and 86 (20.6%) were children under 15 years of age at the time of identification.

**Table 2:** Characteristics of MDR-TB contacts

Characteristic	*n*	%
Total contacts	417	
Age group		
0–14 years	86	20.6
15–24 years	65	15.6
25–34 years	65	15.6
35–44 years	67	16.1
45–54 years	49	11.8
55–64 years	50	12.0
65 year and above	27	6.5
Missing	8	1.9
Median age	32	
Mean age (sd)	34.0 (20.0)	
Gender		
Male	189	45.3
Female	223	53.5
Missing	5	1.2
Type of contact		
Household contact	292	70.0
Non-household contacts	125	30.0

### Screening practices

Of the 417 eligible contacts, 325 (77.9%) participated in the first screening (Fig. 1). Of the remaining 92 contacts, 70 could not be traced and 22 refused (including 3 household and 19 non- household contacts). 160 participated in the second screening including 137 who had also been screened in the first screening. The remaining 188 contacts refused to be screened for second time. The proportions of household contacts and non-household contacts participating in the first screening were 248/292 (84.9%) and 77/125 (61.6%) respectively. The proportions were lower in the second screening: 127/292 (43.5%, *p* < 0.001) and 33/125 (26.4%, *p* < 0.001), respectively. The participation of female contacts in the first screening was higher than for males, 186/223 (83.4%) versus 135/189 (71.4%; *p* = 0.004) (see Additional file 2 for more detailed table). There were no apparent differences in the proportion of contacts participating in the screening by age group (see Additional file 2).

**Fig. 2:**
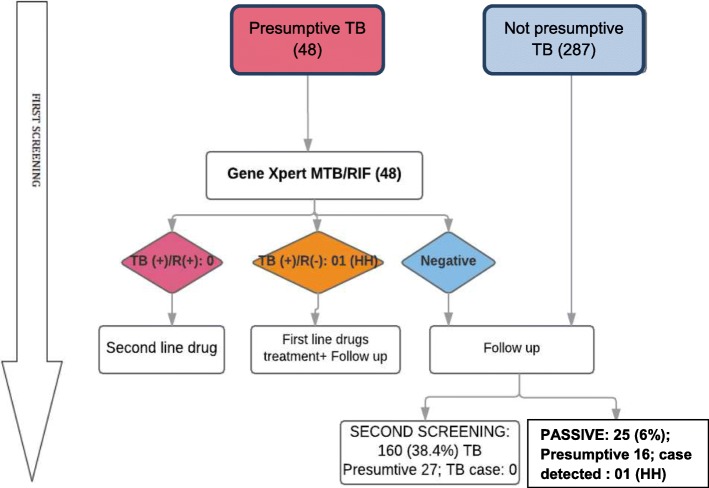
Flow chart of TB screening practice (part B). TB+/R(+): MTB detected with Rifampicin resistance. TB+/R(−): MTB detected without Rifampicin resistance. HH: Household contact

Upon first screening 36/325 (11.1%) contacts interviewed were clinically diagnosed as presumed TB. Chest X-rays were performed for 299 contacts, including 10 with clinically presumed TB, of whom an additional 12 (4.0%) had an abnormal chest X-ray suggestive for TB (Fig. 1). Xpert testing was performed for the total of 48 presumed TB cases identified by clinical assessment and/or by chest X-ray. We detected one drug-susceptible TB case and no rifampicin-resistant/MDR-TB case from the first active screening of contacts (Fig. 2).

Among 160 contacts assessed in the second active screening, twenty-seven (including 3 contacts who also participated in the first screening) had presumptive TB by interview and/or chest X-ray. Xpert MTB/RIF testing detected no TB case. From passive screening, a two-year old child, whose father was an index patient, was diagnosed with TB meningitis but not as part of our study (Fig. 2). This child was not identified as presumed TB in the first screening. She was taken by her parents to the Vietnam national children hospital for diagnosis and not to the district coordinator when later having fever, cough and loss of consciousness.

### Social network analysis

**Fig. 3:**
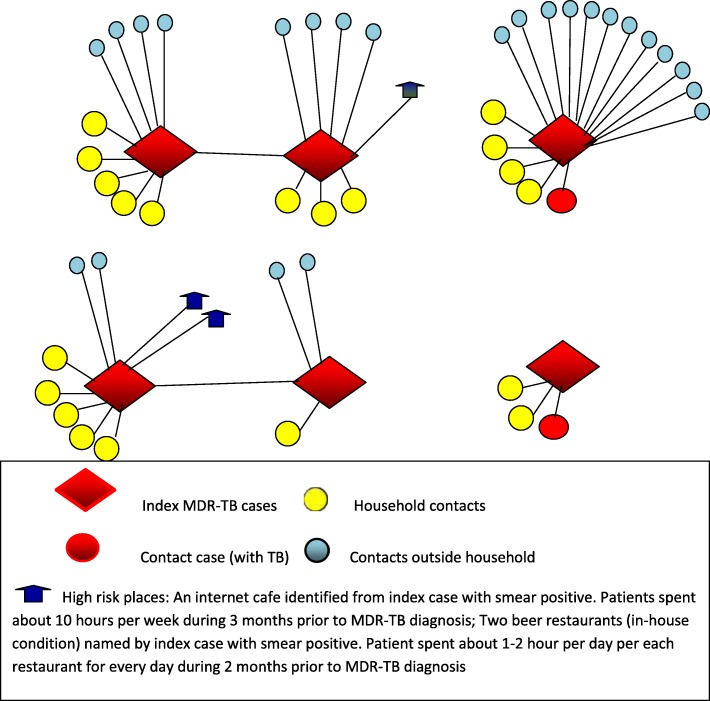
Social network of MDR-TB cases with identified links among index cases and TB cases detected among contacts

The median number of eligible contacts per index patient was 3 (IQR: 3–6). These median numbers were 3 (IQR: 2–4) among household contacts and 2 (IQR: 1–4) among non-household contacts. Index patients named 35 places, of which 17 were identified as high-risk places including 3 workplaces (1 vocational school, 1 private tailoring company, 1 grocery store), 3 internet café’s, 2 hair salons and 9 restaurants. Only 1/17 (5.9%) high-risk place (vocational school) agreed to cooperate. One presumed MDR-TB case was identified based on clinical diagnosis among 15 people screened who frequented this high-risk place (Fig. 1) but not diagnosed as TB. We found no mutual contact and no mutual place among the MDR-TB index cases. Two additional TB cases were detected among household contacts (including the confirmed drug-susceptible case and the child with unknown drug resistance status). Moreover, two pairs of index cases (four patients) were found to be contacts of each other (diagnosed before study started; Fig. 3). No genotyping was done to look at genetic relatedness.

## Discussion

We conducted social network analysis to be able to detect more MDR-TB cases than through passive case finding. Enrolling 99 MDR-TB cases and their contacts did not reveal new MDR-TB cases. One child with (probable) MDR-TB meningitis was missed by our study. Links between MDR-TB cases were found in two instances but did not lead to the detection of new cases. Only one of seventeen high-risk places agreed to participate in the screening, resulting in one additional presumed MDR-TB case identified.

The likely reasons why we did not detect additional MDR-TB cases was limited participation of contacts in TB screening. Participation was reasonable (~ 80%) in the first screening, but dropped considerably to ~ 40% at the second screening. Observation from discussions with staff suggest that participation may have been poor due to the following elements:1) perceived stigma among patients, contacts and high risk places and reluctance to cooperate and to reveal correct contact information, 2) low awareness about TB and its transmission, especially among contacts with low levels of education or contacts belonging to vulnerable groups such as drug users, and 3) participation in the second screening may not have been perceived as in their interest if they were busy and not diagnosed with TB from the first screening.

Furthermore, the relatively short follow-up period in our study of 6 months may be another reason. Studies have shown that active TB usually develops within five years after initial infection [13–15], and predominantly (45%) especially in the first year [16]. The median time from infection to symptoms in secondary cases is estimated to be 1.3 years [16]. Household contacts of MDR-TB patients are considered to be at higher risk to get infected than household members of drug-susceptible TB cases [17, 18]. This is because, even though MDR-TB isolates are usually less transmissible [19], family members of MDR-TB cases tend to have been exposed for a longer duration due to delays in correct treatment initiation[17, 19]. Therefore, contact investigation is useful for early case detection and treatment to reduce transmission of MDR-TB [4, 18].

The pick-up rate for MDR-TB cases may also increase by improving the sensitivity of our diagnostic approach. Future diagnostic approaches should consider: (i) to ensure the quality of sputum and chest X-ray, (ii) expanding TB clinical assessment criteria to any cough and other tuberculosis-related symptoms like chest pain, weight loss, lack of appetite, weakness or fatigue, chills, fever and night sweats (iii) including MTB culture with higher sensitivity [20] as an add-on test following Xpert result, (iv) using multiple rather than a single specimen, to increase the diagnostic yield of Xpert MTB/ RIF [20]. However, resources in low and middle-income countries (LMICs) are generally limited and therefore it may not be feasible to implement all these recommendations.

A limitation of our study using Xpert MTB/RIF is that only TB and rifampicin resistance is diagnosed as an indicator for MDR-TB [20]. In Vietnam we generally also perform culture and additional sensitivity testing of drugs included in first and second line regimens to confirm MDR-TB and tailor treatment.

There is a need to develop a system to identify and manage contacts of MDR-TB cases better, including providing of adequate instructions, and possibly screening. We recommend to use a simpler questionnaire rather than a comprehensive social network approach. This is a more efficient and likely more cost-effective means for MDR-TB case detection in Vietnam and other low and middle-income countries. Information about household contacts and those who have the most frequent contact with patients such as close friends and colleagues should be collected. Depending on available resources, screening may start with a clinical assessment to determine if the person has TB-related symptoms, followed by chest X-ray and Gene Xpert MTB/RIF. This should be combined with health education, i.e. inform contacts with what symptoms they need to come for TB screening.

Health education about TB, MDR-TB and its transmission among the general population should be more focused, and results of this study may help in prioritizing risk groups. It is needed to enhance awareness among contacts of MDR-TB and their compliance with screening programmes. Particular attention should be paid to enhance screening of non-household contacts as some studies show the incidence of TB among these contacts to be higher compared with household contacts [5–7]. Furthermore, we found a lower screening participation of male contacts in our study, which is in line with findings from our national prevalence survey. Therefore, more efforts are needed to find male tuberculosis patients [21].

Currently, about 50% of the estimated MDR-TB cases in Vietnam have not been previously treated, reflecting significant transmission of MDR-TB among contacts [20–24]. However, the routine case finding strategy for detection of MDR-TB during our study period mainly focused on previously treated TB cases Additional file 1 [10], with only 14% of MDR-TB patients diagnosed being treatment-naive. It is important for Vietnam to pay more attention to management of MDR-TB among new cases including close monitoring of MDR-TB contacts. Given the low yield of MDR-TB case detection from our study, beyond improving contact investigation, other potential groups should be considered to address 50% of undetected MDR-TB burden in Vietnam. Furthermore, diagnostic screening strategies should be enhanced. Approaches can be applied depending on the resources available as follows: microbiological testing by Gene Xpert MTB/RIF for (i) all newly detected TB patients including smear positive and negative (ii) presumptive TB cases who had/have contact with MDR-TB patients. These contacts can be identified by healthcare workers through interviewing TB presumptive cases who come to their health facility for health check up, and (iii) all TB presumptive.

While MDR-TB can be cured, social barriers to MDR-TB treatment could be an important factor that needs to be taken into account when designing and implementing a contact tracing program [17]. Home visits by contact investigators are an effective method for interviewing household contacts and encouraging them to be assessed for TB [4]. By visiting index patients and their household contacts, the investigator is able to observe the housing conditions, perform an environmental assessment for infection control measures, and discuss and evaluate the risk of exposure, as well as provide counseling to household contacts on symptoms suggestive of TB and when and where to seek health care and social support [4].

Even though we did not find any new MDR-TB case directly through our social network analysis, this approach may still be worth consideration if the key limitations of our study are addressed. The screening process should be simplified, well organized, to increase the participation of contacts, extend the time of follow-up of contacts, and improve diagnostic screening strategy. Given that the low participation rate in our study may have limited case detection, it is recommended to expand health education on transmission of TB and MDR-TB among contacts, reduce stigma attached to TB, improve communication skills of health staff, and increase staff resources to trace contacts and get them involved in the screening.

## Conclusion

In this study of nearly 100 MDR-TB index cases we were not able to find new MDR-TB cases using household contact screening and social network analysis within a follow-up period of 6 months. Screening of identified contacts was complicated by refusals. More staff resources may be needed and better communication skills and community awareness, collaboration of non NTP health facilities is needed to enhance participation and improve MDR-TB case detection.

## Additional files

Additional file 1: Policy and performance of the National TB Control Programme of Viet Nam. Notification and enrollment for treatment of MDR-TB from 2009 to 2014. Policy recommended by the NTP Guidelines for detection of MDR-TB. (DOCX 39 kb)

Additional file 2: Additional information for Methods and Results Sections. Sample size calculation. Modification description of questionnaire and the completeness of responses. Operational definitions and definition of patient category. Screening among contacts by gender, age and contact type. (DOCX 49 kb)

## Abbreviations

CRF: Case report form
MDR-TB: Multidrug-resistant tuberculosis
NTP: National Tuberculosis Control Programme
SNQ: Social network questionnaires
WHO: World Health Organization
