# Supplementary material for: Active contact tracing beyond the household in multidrug resistant tuberculosis in Vietnam: a cohort study
Source: BMC Public Health. 2019 Feb 28;19:241. doi: 10.1186/s12889-019-6573-z (PMC6394002; doi:10.1186/s12889-019-6573-z)
Supplement: Supplementary file 1 — Policy and performance of the National TB Control Programme of Viet Nam. Notification and enrollment for treatment of MDR-TB from 2009 to 2014. Policy recommended by the NTP Guidelines for detection of MDR-TB. (DOCX 39 kb) [file 12889_2019_6573_MOESM1_ESM.docx]

**POLICY AND PERFORMANCE OF THE NATIONAL TB CONTROL PROGRAMME OF VIETNAM**

**Notification and enrollment for treatment of MDR-TB from 2009-2014 (For reference 3)**

| Year | 2009 | 2010 | 2011 | 2012 | 2013 | 2014 |
| --- | --- | --- | --- | --- | --- | --- |
| No. of MDR-TB diagnosed | 278 (*) | | 697 | 774 | 997 | 1790 |
| No. of patients enrolled for treatment | 101 | 97 | 578 | 713 | 948 | 1532 |
| MDR-TB, estimates among notified cases | 3500 | 3500 | 3700 | 3800 | 5100 | 5100 |
| % enrollment among estimated number | **3%** | **3%** | 16% | 19% | 19% | 30% |

*(*) Total number of MDR-TB diagnosed for two years ( 2009 and 2010)*

**Policy recommended by the NTP Guidelines for detection of MDR-TB (For reference 10)**

The National Guideline for Programmatic Management of Drug Resistance Tuberculosis (page 15-16) states that*“individuals who have close contact with multiple-drug resistant TB case are at high risk of developing drug-resistant TB. There is a need to pay more attention to manage contacts of MDR-TB patients properly”*. However, there no clear instructions provided by the Guideline to support the implementation of this recommendation.

With regard to risk groups to be screened for MDR-TB as recommeded by the Guideline, the National TB Control Programme of Vietnam only focuses on specific risk groups, mainly targeted at previously treated cases:

- Patients who have failed from category I regimen for new cases.
- Patients who have failed from category II regimen for retreatment.
- Patients who return after loss to follow-up from previous TB treatment and have a smear positive of the sputum examination
- TB presumptive patients (with signs and symptoms of TB) who have contact with MDR-TB patients, or are at high risk for DR-TB (health care worker, laboratory technician, people living in congregate settings such as jails, rehabilitation centers; people living with HIV/AIDS).
