# Supplementary material for: Active contact tracing beyond the household in multidrug resistant tuberculosis in Vietnam: a cohort study
Source: BMC Public Health. 2019 Feb 28;19:241. doi: 10.1186/s12889-019-6573-z (PMC6394002; doi:10.1186/s12889-019-6573-z)
Supplement: Supplementary file 2 — Additional information for Methods and Results Sections. Sample size calculation. Modification description of questionnaire and the completeness of responses. Operational definitions and definition of patient category. Screening among contacts by gender, age and contact type. (DOCX 49 kb) [file 12889_2019_6573_MOESM2_ESM.docx]

## ADDITIONAL INFORMATION FOR METHODS AND RESULTS SECTIONS

## Sample size calculation

It was estimated that one index MDR-TB case would have approximately 3 eligible contacts. It was expected that 75% of contacts will be screened. It was assumed that 10% of the contacts will be symptomatic of which 15% will have TB and 60% of TB will be MDR. Therefore, the yield of contact tracing by SNQ to MDR-TB case detection for 100 index cases was estimated to be 2.0% (2 cases per 100 index cases). The minimum sample size we needed in order to detect the estimated yield of active case finding of 2cases per 100 index cases with 95% CI significantly different from 0 is 100 as shown in table bellow

| Index patients recruited (n) | Estimated  eligible contacts enrolled (n)* | Contacts with active TB (n)** | Expected point estimate for proportion of contacts with TB (%) *** | Estimated 95% confidence limits | |
| --- | --- | --- | --- | --- | --- |
|  |  |  |  | lower | Upper |
| 200 | 600 | 4 | 2.0 | 0.6 | 5.0 |
| 200 | 600 | 3 | 1.5 | 0.3 | 4.3 |
| 100 | 300 | 3 | 3.0 | 0.6 | 8.5 |
| 100 | 300 | 2 | 2.0 | 0.2 | 7.0 |
| 100 | 300 | 1 | 1.0 | 0 | 5.5 |
| 50 | 150 | 3 | 6.0 | 1.3 | 16.5 |
| 50 | 150 | 2 | 4.0 | 0.4 | 13.7 |
| 50 | 150 | 1 | 2.0 | 0 | 10.6 |

*Based on assumption of 3 contacts screened per index patient. **Assumption for number of contacts diagnosed as active TB. *** Number of patients detected by SNQ divided by number of index cases

**Modification description of questionnaire and the completeness of responses**

The social network questionnaire used in reference 11 was modified and contextualized for our study. Modifications were made as follows:

Modification of questionnaire structure:

The structure in our questionnaire was rearranged into four sections instead of eight sections as in reference 11:(i) identifying data, (ii) medical history and onset symptoms, (iii) social network, and (iv) places of social aggregation. This is a way to group similar information into the same sections, which facilitates the collection of information more conveniently

In addition to the main form for gathering general information in four sections, detailed information about each contact, each location is guided to separate forms (either“ contact information” form or “places information” form) to gather more detailed information.

Content of each section were contextualized and/or expanded as necessary

We did not collect information that is not applicable to the Vietnamese situation (e.g. postal code for identifying data, residence condition in detail as how many toilets, presence of heating or hot water). Instead, we selected information that is more appropriate such as whether the places are enclosed spaces (restricted area equivalent to a domestic room, the same indoor living space), and what place category they were (workplace, place of social aggregation, treatment ward, hospital, sleeping location/residence, etc)

Regarding places of social aggregation we were also interested in places where patients worked or frequently travelled to, even including bus lines that patients took

Method to complete questionnaire:

Information was not only collected directly during patient interviews, but also from other databases like the National TB Control Programme (e.g. information for physical description, patient category and laboratory testing)

To help the recall of the patient, several prompts were used during interview. For example, if a patient could not remember the onset time of symptoms, we started back a few months and used specific seasons or national holidays. To ensure the maximum number of contacts the patient could name, patients were not only asked to name people they met; but also to check the contact list in their telephone and name who they saw when and where.

- In order to know more about the possible places visited, we trained the interviewer to let the patient speak first without providing the locations of interest to see if they were named. This was followed by prompts (homes of friends and acquaintances, shops, restaurants, and so on).

The completeness of responses to the questionnaire was very good: none of the 99 patients refused to answer any of the questions posed in the questionnaire, and all questions were filled out. However, of the 417 contacts, eight had no gender information and five had no age information (as shown in Table 2) provided.

**Operational definitions**

**MDR-TB presumptive case:** In this study, a MDR-TB presumptive case referred to any MDR-TB contact with (i) clinical symptoms suggestive of TB or (ii) abnormal findings consistent with TB on the chest X-ray (CXR) or (iii) TB patients who were on current first-line anti-TB treatment.

**Multidrug-resistant tuberculosis (MDR-TB) case**: defined as TB caused by strains of *Mycobacterium tuberculosis* that are resistant to at least isoniazid and rifampicin. In this study, patients who are tested by Gene Xpert MTB/RIF and are positive for rifampicin-resistant TB were also classified as MDR-TB (because more than 96% of rifampicin resistance in Vietnam is also resistant to isoniazid[1]).

**Index MDR-TB case**: rifampicin-resistant or MDR-TB patient who was diagnosed and enrolled into treatment by the routine care system.

**Household contact**: all household members of the MDR-TB patients, including children, who were present in the household at least 4 hours a day, for at least 14 days, or a cumulative total average period of at least 8 hours per week for at least 8 weeks during 3 months leading up to the time of TB diagnosis.

**Close contact outside household:** any individual apart from household members who also had frequent and prolonged contact with the MDR-TB patients (i) in indoors environment during 3 months leading up to the time of TB diagnosis, and (ii) spent at least 4 hours a day, for at least 14 days, or a cumulative total average period of at least 8 hours per week for at least 8 weeks together with the patient indoors [2], [3]

**Mutual contact**: a contact that was named by at least 2 confirmed MDR-TB patients.

**Mutual place**: a place that was named by at least 2 confirmed MDR-TB patients.

**High risk place:** The indoors environment where the MDR-TB index case spent an average of at least 4 hours a day for at least 14 days, or a cumulative total average period of at least 8 hours per week for at least 8 weeks in the 3 months prior MDR-TB diagnosis.

**Eligible place:** included mutual places and high-risk places.

**Eligible contact:** Included (i) household contacts, (ii) close contacts outside the household, (iii) mutual contacts, (iv) people who frequently visited the eligible places during 3 months leading up to MDR-TB diagnosis and found to be TB presumptive by primary interview.

**Patients category:**

- New TB case: TB patients who had received no or less than one month of TB treatment
- Previously treated cases: TB patients who had received TB treatment previously with treatment outcome declared.
- Non converters: TB patients whose sputum had not converted (from positive to negative) or had reverted (from negative to positive) after 2 months of TB treatment
- Others: Other TB patients including TB patients who had previously treatment for TB in the private sector with unknown outcome, and/or patients who had been diagnosed as smear-negative in a previous TB treatment course, but were diagnosed as smear-positive currently.

**Definition of patient category**

- **New:** A patient who has received no anti-TB treatment before or only used anti-TB drugs for less than one month
- Non-converters of first line drug for new cases: TB patients whose sputum did not convert after 2 and 3 months of regimen with first line drug for new cases (cat I regimen).
- Non-converters of first line drug for retreatment cases: TB patients whose sputum did not convert after 3 months of regimen with first line drug for retreatment cases (cat II regimen).
- Previously treated cases include the following sub-categories of patients
  - Treatment after failure of Category I (for new TB cases) and/or Category II (for retreatment cases). Failure is defined as sputum smear positive at five months or later during treatment.
  - Relapse of Category I and/or Category II. Relapse is defined as a patient whose most recent treatment outcome was “cured” or “treatment completed”, and who is subsequently diagnosed with bacteriological positive TB by sputum smear microscopy or culture
  - Treatment after default of Category I and/or Category II. Default is defined as a patient who returns to treatment, bacteriological positive by sputum smear microscopy or culture, following interruption of treatment for two or more consecutive months.
- **Others:** Other TB patients, including TB patients who were previously treated for TB in the private sector with unknown outcome, and/or patients who were diagnosed as smear negative in previous TB treatment course, but are currently smear positive.

**REFERENCES**

[1] Nhung. N. V, Hoa.N. B, Sy.D. N, Hennig.C. M, Dean.A. S. The Fourth National Anti-Tuberculosis Drug Resistance Survey in Viet Nam. Int J tuber Lung dis. 2015; 19(6): 670–5

[2] World Health Organization. Recommendations for investigating contacts of persons with infectious tuberculosis in low and middle-income countries. WHO/HTM/TB/2012.9. Geneva, WHO, 2012.

[3] Greenaway C, Palayew M, Menzies D. Yield of casual contact investigation by the hour. Int J Tuberc Lung Dis 2003; 7: Suppl. 3, S479–S485

**Screening among contacts by gender, age and contact type**

|  | | | Eligible contacts | First screening | Second screening |
| --- | --- | --- | --- | --- | --- |
| Gender | Male | Number | 189 | 135 | 69 |
|  |  | % within gender |  | 71.4% | 36.5% |
|  | Female | Number | 223 | 186 | 90 |
|  |  | % within gender |  | 83.4% | 40.4% |
|  | total |  | 412 | 321 | 159 |
| Age group | 0-14 | Number | 86 | 75 | 45 |
|  |  | % within age group |  | 87.2% | 52.3% |
|  | 15-24 | Number | 65 | 53 | 16 |
|  |  | % within age group |  | 81.5% | 24.6% |
|  | 25-34 | Number | 65 | 51 | 27 |
|  |  | % within age group |  | 78.5% | 41.5% |
|  | 35-44 | Number | 67 | 51 | 31 |
|  |  | % within age group |  | 76.1% | 46.3% |
|  | 45-54 | Number | 49 | 32 | 14 |
|  |  | % within age group |  | 65.3% | 28.6% |
|  | 55-64 | Number | 50 | 40 | 18 |
|  |  | % within age group |  | 80.0% | 36.0% |
|  | 65 above | Number | 27 | 21 | 7 |
|  |  | % within age group |  | 77.8% | 25.9% |
|  | Total |  | 409 | 323 | 158 |
| Type of contacts | Household contact | Number | 292 | 248 | 127 |
|  |  | % within contact type |  | 84.9% | 43.5% |
|  | Outside household contacts | Number | 125 | 77 | 33 |
|  |  | % within contact type |  | 61.6% | 26.4% |
|  | Total |  | 417 | 325 | 160 |
